# Implementing stakeholder-informed research in the substance abuse treatment sector: strategies used by Connections, a Canadian knowledge translation and exchange project

**DOI:** 10.1186/1747-597X-9-21

**Published:** 2014-05-29

**Authors:** Joanna Henderson, Wendy Sword, Alison Niccols, Maureen Dobbins

**Affiliations:** 1 Centre for Addiction and Mental Health, Department of Psychiatry, University of Toronto, 80 Workman Way, Toronto M6J 1H4, Canada; 2 School of Nursing, McMaster University, Hamilton, ON L8N 3Z5, Canada; 3 Department of Psychiatry and Behavioural Neurosciences, McMaster University, McMaster Children's Hospital-Chedoke Site, Holbrook Building, Hamilton, ON L8N 3Z5, Canada

**Keywords:** Integrated knowledge translation, Stakeholder-informed research, Addiction services, Women, Service providers, Policy-makers

## Abstract

**Background:**

Researcher-stakeholder collaboration has been identified as critical to bridging research and health system change. While collaboration models vary, meaningful stakeholder involvement over time (“integrated knowledge translation”) is advocated to improve the relevance of research to knowledge users. This short report describes the integrated knowledge translation efforts of Connections, a knowledge translation and exchange project to improve services for women with substance abuse problems and their children, and implementation barriers and facilitators.

**Findings:**

Strategies of varying intensities were used to engage diverse stakeholders, including policy makers and people with lived experience, and executive directors, program managers, and service providers from Canadian addiction agencies serving women. Barriers to participation included individual (e.g., interest), organizational (e.g., funding), and system level (e.g., lack of centralized stakeholder database) barriers. Similarly, facilitators included individual (e.g., perceived relevance) and organizational (e.g., support) facilitators, as well as initiative characteristics (e.g., multiple involvement opportunities). Despite barriers, Connections’ stakeholder-informed research efforts proved essential for developing clinically relevant and feasible processes, measures, and implementation strategies.

**Conclusions:**

Stakeholder-researcher collaboration is possible and robust integrated knowledge translation efforts can be productive. Future work should emphasize developing and evaluating a range of strategies to address stakeholders’ knowledge translation needs and to facilitate sustained and meaningful involvement in research.

## Background

Researcher-stakeholder collaborations are considered critical for addressing continuing gaps between research and health system change [1]. Following in the tradition of participatory research, it has been argued that for system change to take place knowledge-users, including front-line practitioners, organizational decision-makers, policy-makers, service users, and other stakeholders need to be meaningfully involved with the research process from project development initiation, and have genuine opportunities to influence project design, processes, and outputs [1]. Often referred to as “integrated knowledge translation”, (iKT) this approach has received increased funding agency attention and many grants require applicants to include iKT plans [1]. Recent iKT attention stems from acknowledgement that barriers to implementing evidence-based practice arise from research innovation characteristics, including its perceived relevance to and compatibility with existing practice, as well as its feasibility and effectiveness with ‘real’ clinical populations (for a review see [2]). By integrating stakeholders into the research process, it is expected that the feasibility and relevance of the knowledge and products generated will be enhanced [1,3] and uptake improved. Indeed, emerging evidence supports the potential utility of iKT in developing knowledge and products perceived as relevant [4] and in promoting uptake of evidence-informed practices and policies [5]. Accordingly, this article describes the use of iKT strategies throughout the design and implementation of a Canadian knowledge translation initiative focused on enhancing the use of evidence-based practice in services for women with substance use issues and their children.

Notably, while there has been some early evidentiary support for iKT, there is a continuing need to operationalize iKT [6]. Kothari and Wathen define iKT as “the development of a relationship between academic researchers and practitioners and/or policymakers for the purposes of collaboratively engaging in a mutually beneficial research project or programme of research” [7]. While this highlights some key iKT aspects, it does not identify which strategies and activities constitute iKT, nor how to achieve its goals. Some iKT-related activities that have been proposed include information sharing, meetings, and joint task completion, including developing research questions and designs, data collection planning and implementation, data analysis and interpretation, and recommendation generation [7-11]. These activities overlap with the aims of participatory research, although iKT is more focused on integrating the end users of the knowledge being created as opposed to individuals affected by the issue under investigation more broadly (e.g., patients) and participatory research requires stakeholder participation throughout whereas a range of iKT activities can be considered acceptable depending on what is considered necessary to move the research to practice [1,12].

Like participatory research and other forms of community-based research, however, iKT efforts can be hampered by many collaboration challenges. For example, the time, effort and resources required to meaningfully participate in collaborative research can exceed what is feasible, especially for individuals for whom research is not considered part of their typical role, such as service providers [7,10,11]. Also, differences in expected pace and timelines, particularly the short, demanding timelines of some grant applications and the slow pace of results generation can lead stakeholders to feel their needs have not been met [10,11,13,14]. Moreover, stakeholders may enter collaborations with priorities, goals, and expected benefits that differ substantially, and which may be met to varying degrees. To the extent that priorities, goals, and expected benefits are unmet, stakeholders may reduce or restrict their further involvement, limiting the potential benefits originally intended by the collaboration [7]. Organizational factors also can impact collaboration success. Staff turnover, organizational culture, especially differing views of ‘evidence’, and variations in accountability and reward systems can hamper functioning of stakeholder-researcher partnerships [7,10,11,13-18].

Connections is a research program dedicated to developing and evaluating knowledge translation and exchange (KTE) strategies to facilitate evidence-informed decision-making in women’s services for substance abuse issues [19]. Given the Connections Research Team’s interest in developing relevant and feasible strategies for enhancing evidence-based practice, the use of iKT strategies was considered essential. In addition to expanding knowledge about factors affecting KTE success in the addictions sector, Connections conducted a multi-site feasibility study of a knowledge broker intervention to enhance evidence-informed decision-making. From the outset, the Connections Research Team emphasized iKT and over the 5-year initiative multiple iKT strategies were utilized. In this short report, we describe the strategies used, barriers and facilitators, and implications for future iKT efforts.

## Methods

### Connections

The Connections Research Team includes 13 multidisciplinary researchers from across Canada with varying areas of expertise, including KTE, health (women’s, children’s), addictions, treatment evaluation, biostatistics, research methodology, qualitative research, and network analyses. During the grant development phase a National Advisory Committee of key stakeholders, including individuals with lived experience, was developed to provide ongoing consultation. Our primary iKT goal was to enhance our shared understanding of stakeholders’ KTE preferences, evidence-informed decision-making processes, service integration, cross-sectoral networking (e.g., women’s addictions, adult mental health, children’s services), and the clinical needs of women and their children. We aimed to achieve this by developing a sense of belonging to a collaborative inter-professional community dedicated to improving access to evidence-informed care within the addictions sector for women with substance abuse problems and their children.

### Procedure

Executive directors, program managers, and front line staff from addiction agencies serving women and individuals with lived experience, administrators, policy makers, and representatives from other relevant local, provincial, and national organizations were invited to participate in a range of opportunities grouped into three main strategic approaches: 1) Information sharing (e.g., website, media press releases, conferences, and other presentations); 2) Informal input-seeking (e.g., individual consultations, requests for written feedback); and 3) Formal input-seeking (e.g., surveys, semi-structured individual interviews, focus groups, networking events, National Advisory Committee meetings, and Research Team meetings).

## Findings

Over 2000 stakeholders participated via two surveys (*n* = 1800), three focus groups (*n* = 24), four networking events (*n* = 111), five National Advisory Committee meetings (*n* = 25), and three studies involving individual interviews (*n* = 81). In addition, the Connections’ website received over 10,000 hits, we published 14 papers, several hundred people attended our 24 conference presentations and/or provided input in response to email consultation requests. We also trained 14 students.

### Barriers

Through team member observation of patterns of participation, and stakeholder feedback that was either formally sought (e.g., at networking events) or informally received (e.g., individual email response to a request), we noted a number of barriers to stakeholder participation. At the individual level, stakeholder involvement was affected by lack of time, clinical demands, lack of interest, and lack of perceived expertise to make valuable contributions. At the organizational level, resource constraints, agency and funder priorities, and staff turnover presented barriers to participation. At the system level, a lack of a centralized stakeholder database to facilitate identification of relevant stakeholders hampered iKT efforts.

### Connections’ response to barriers

Connections’ efforts to ensure wide stakeholder participation led to the development and implementation of specific strategies to address barriers. For example, in order to address challenges associated with resource limitations, Connections provided free capacity building and networking events, provided financial support for travel to bring stakeholders together, and provided backfill support to agencies for staff participation. In addition, in response to stakeholder input, Connections held some of their events to coincide with other events that stakeholders typically attended, e.g., national substance abuse conferences, reducing the travel burden and leveraging existing networking opportunities. Lastly, Connections provided a range of opportunities for stakeholder involvement, reflecting the diversity of interest levels and capacities for participation expressed by stakeholders. The selected strategies ranged in the time, effort, and resources required to implement and to participate, the number of stakeholders who could feasibly be involved, and the breadth and depth of information that could be gathered. Surveys, for example, provided a relatively low resource-intense mechanism for gathering a broad range of information from a wide range of stakeholders at repeated points in time. Networking events and interviews, on the other hand, afforded opportunities to gather detailed information about a narrower range of issues from a smaller stakeholder group.

### Facilitators

Like with barriers, formal and informal stakeholder feedback and team observations revealed a number of facilitators of stakeholder participation. At the individual level, perceived clinical need and relevance of improving services to women with substance abuse issues and their children was considered to be a key driver to stakeholder involvement. For example, stakeholders viewed Connections as promoting the collaboration and networking required to improve service delivery to women whose needs are often complex and multi-faceted, and as enhancing integration of research knowledge and practice experience, which they considered essential to improving outcomes [20]. At the organizational level, resource support provided by Connections to agencies, including free capacity-building events, travel sponsorship, online literature database access, and back-fill funding, provided much needed support to facilitate participation. At the iKT intervention level, stakeholders identified Connections’ flexible multifaceted approach with opportunities for involvement that varied in intensity and resource requirements, its utilization of local experts and leads, its multi-disciplinary team and advisory committee, and its overall collaborative approach to be essential components promoting stakeholder participation.

### Impact

The impact of stakeholder involvement on Connections’ research was substantial. Resulting from stakeholder input, Connections’ originally proposed processes, measures, and interventions were kneaded into stakeholder-informed processes, measures, and interventions considered highly relevant and feasible to clinical settings. For example, the planned KTE intervention study did not originally include a knowledge broker but in response to stakeholder feedback, we altered the planned intervention to include a 12-month trial of a Connections’ knowledge broker providing ‘on-the-ground’ support to enhance evidence-informed decision-making knowledge, attitudes, and skills to 15 agencies across Canada. Further, the study did not originally include providing subscriptions to online databases but in response to stakeholder discussions about their access to evidence, our intervention expanded to include providing subscriptions to online databases of research evidence to each participating agency. Moreover, clinically relevant research questions raised by stakeholders and for which sufficient evidence does not exist have become the basis of further research funding proposals.

Notably, different iKT strategies were found to have varied effects. Among the formalized approaches, those primarily quantitative strategies that reached a larger number of stakeholders across a wider range of areas of inquiry (e.g., surveys) resulted in broader stakeholder representation and greater diversity of perspectives. Those strategies that were narrower in scope, such as individual interviews, provided better issue representation with specific areas of inquiry explored in detail but fewer perspectives could feasibly be explored. In combination, however, these strategies allowed the team to forge a greater understanding of the range and complexity of KTE-related issues that needed to be addressed to enhance research on evidence-informed decision making among service providers working with women with substance abuse issues.

Although many stakeholders had sporadic or single episode involvement, other stakeholders had ongoing involvement over the 5-year project. Indeed, for some stakeholders, their roles shifted from initial low intensity involvement (e.g., providing ad hoc consultation) to higher intensity roles, such as local lead at a study intervention site. These experiences highlighted the potential for long-lasting meaningful stakeholder-researcher collaborations to function despite the barriers, and emphasized their role in promoting successful KTE.

### Limitations

We did not set out to systematically study our iKT activities and as a result, our methods for tracking barriers and facilitators, particularly those identified through informal feedback, were somewhat limited. In addition, the stakeholders who participated were not necessarily representative of all members of their stakeholder groups. Future studies of iKT efforts should endeavor to gather information systematically about both participating and non-participating stakeholders.

### Implications

Connections’ iKT experiences underscore the need to develop and evaluate a broad range of iKT strategies, and suggest that the inclusion of a wide range of activities within the iKT definition e.g., [7-11] is warranted. As has been suggested by Kothari & Wathen (2013), limited stakeholder time and resources pose significant collaboration challenges. Despite these and other barriers, however, iKT is feasible and for Connections, iKT contributed meaningfully to project processes and outcomes. Multi-pronged approaches that offer flexible opportunities for involvement and that respond to stakeholders’ needs, such as those used by Connections, appear essential for promoting sustainable iKT. Moreover, resources dedicated to supporting meaningful and sustained stakeholder involvement are critical if enhanced researcher-stakeholder collaboration is to become commonplace and maximally effective [7]. Indeed, service funders, like researcher funders, may need to consider allocating resources to support stakeholder-researcher partnerships in the pursuit of relevant and usable clinical knowledge.

Notably, limited attention has been paid to systemically documenting the cost to stakeholders of their collaboration in research. In addition, little information exists about the effects of iKT on stakeholders’ research-related knowledge, attitudes, and practices, as well as researchers’ understanding of ‘real world’ service delivery contexts and integration of stakeholder input into project design and processes. Future research should address these gaps.

## Conclusions

Effective use of iKT strategies is possible and recommended. In order to develop meaningful collaborations, it is suggested that researchers consider potential barriers as well as strategies to overcome barriers and facilitate stakeholder involvement early in the project planning and funding application process. Multi-method approaches for stakeholder involvement must be considered and evaluated. Also, iKT efforts and stakeholder responses must be systematically documented. Lastly, greater attention needs to be paid to understanding which engagement strategies work best for which stakeholders, for what purpose, and with what level of effectiveness.

## Abbreviations

iKT: Integrated knowledge translation; KTE: Knowledge translation and exchange.

## Competing interests

The named authors declare that they have no competing interests.

## Authors’ contributions

JH contributed to the planning and implementation of the stakeholder informed research protocol and drafted the manuscript. WS, AN and MD led the planning and implementation of Connections as well as its stakeholder informed research protocol and helped to draft the manuscript. The Connection Research Team contributed to the planning and implementation of the stakeholder informed research protocol. All named authors read and approved the final manuscript.

## References

[B1] Graham ID Tetroe JM Getting evidence into policy and practice: perspective of a health research funder J Can Acad Child Adolesc Psychiatry 2009 18 46 50 19270848 PMC2651211

[B2] Greenhalgh T Russell J Ashcroft RE Parsons W Why national eHealth programs need dead philosophers: Wittgensteinian reflections on policymakers’ reluctance to learn from history Milbank Q 2011 89 533 563 10.1111/j.1468-0009.2011.00642.x 22188347 PMC3250633

[B3] Henderson J Milligan K Niccols A Thabane L Sword W Smith A Rosenkranz S Reporting of feasibility factors in publications on integrated treatment programs for women with substance abuse issues and their children: a systematic review and analysis Health Res Policy Syst 2012 10 1 13 10.1186/1478-4505-10-1 23217025 PMC3547724

[B4] Martens PJ The right kind of evidence-integrating, measuring, and making it count in health equity J Urban Health 2012 89 925 936 10.1007/s11524-012-9738-y 22772770 PMC3531355

[B5] Harrison MB Graham ID Roadmap for a participatory research-practice partnership to implement evidence Worldviews Evid Based Nurs 2012 9 210 220 10.1111/j.1741-6787.2012.00256.x 22672620 PMC3791554

[B6] Ross S Lavis J Rodriguez C Woodside J Denis JL Partnership experiences: involving decision-makers in the research process J Health Serv Res Policy 2003 8 Suppl 2 26 34 10.1258/135581903322405144 14596745

[B7] Kothari A Wathen CN A critical second look at integrated knowledge translation Health Policy 2013 109 187 191 10.1016/j.healthpol.2012.11.004 23228520

[B8] Bowen S Martens P Demystifying knowledge translation: learn from the community J Health Serv Res Policy 2005 10 203 211 10.1258/135581905774414213 16259686

[B9] Golden-Biddle K Reay T Petz S Witt C Casebeer A Pablo A Hinings CR Toward a communicative perspective of collaborating in research: the case of the researcher-decision-maker partnership J of Health Serv Res Policy 2003 8 Suppl 2 20 25 10.1258/135581903322405135 14596744

[B10] Henderson J Brownlie EB Rosenkranz S Chaim G Beitchman J Integrated knowledge translation and grant development: addressing the research practice gap through stakeholder-informed research J Can Acad Child Adolesc Psychiatry 2013 22 268 274 24223045 PMC3825466

[B11] Lomas J Using ‘linkage and exchange’ to move research into policy at a Canadian foundation Health Aff 2000 19 236 240 10.1377/hlthaff.19.3.236 10812803

[B12] Parry D Salsberg J Macaulay AC A Guide to Researcher and Knowledge-User Collaboration in Health Research 2009 Canadian Institutes of Health Research: Ottawa

[B13] Denis JL Lomas J Convergent evolution: the academic and policy roots of collaborative research J Health Serv Res Policy 2003 8 Suppl 2 1 6 10.1258/135581903322405108 14596741

[B14] Minkler M Community-based research partnerships: challenges and opportunities J Urban Health 2005 82 Suppl 2 ii3 ii12 15888635 10.1093/jurban/jti034 PMC3456439

[B15] Dobbins M Hanna SE Ciliska D Manske S Cameron R Mercer SL O’Mara L DeCorby K Robeson P A randomized controlled trial evaluating the impact of knowledge translation and exchange strategies Implement Sci 2009 4 1 16 10.1186/1748-5908-4-1 19775439 PMC2936828

[B16] Henderson JL MacKay S Peterson-Badali M Interdisciplinary knowledge translation: Lessons learned from a mental health-fire service collaboration Am J Community Psychol 2010 46 277 288 10.1007/s10464-010-9349-2 20857330

[B17] Kothari A Armstrong R Community-based knowledge translation: unexplored opportunities Implement Sci 2011 6 1 6 10.1186/1748-5908-6-1 21645381 PMC3127775

[B18] Lomas J The in-between world of knowledge brokering BMJ 2007 334 129 132 10.1136/bmj.39038.593380.AE 17235094 PMC1779881

[B19] Niccols A Dobbins M Sword W Henderson J Smith P Thabane L Dewit D Lipman E Milligan K Jack S Schmidt L Dooley M Optimizing the health of women with substance use issues and their children[https://www.connectionscanada.ca]

[B20] Connections Learning from the Field: Summary of Fall 2010 Networking Meetings 2010 Hamilton

